# Hormone Therapy to Treat Cardiac Remodeling: Is There Any Evidence?

**DOI:** 10.5935/abc.20160106

**Published:** 2016-07

**Authors:** Luís Alexandre F. Cicchetto, Bertha F. Polegato, Leonardo A. M. Zornoff

**Affiliations:** Departamento de Clínica Médica - Faculdade de Medicina de Botucatu - Universidade Estadual Paulista Júlio de Mesquita Filho - UNESP, Botucatu, SP - Brazil

**Keywords:** Hormone Replacement Therapy, Heart Failure, Ventricular remodeling, Testosterone, Thyroid Hormones

Cardiac remodeling can be defined as a set of molecular, cellular and interstitial cardiac variations, which manifest clinically as changes in heart size, mass, geometry and function in response to certain aggressions. That process has bad prognosis, because it is associated with ventricular dysfunction progression and malignant arrhythmias.^1^ Different therapeutic strategies have been assessed to prevent, or at least attenuate, cardiac remodeling.^2,3^ Some treatments, such as those using angiotensin-convert-enzyme inhibitors, angiotensin II receptor blockers, beta-blockers, and aldosterone antagonists, have been well established. Other treatments, despite their pathophysiological potential, are still being studied.^4^ Of those, hormone therapy stands out, particularly with testosterone, thyroid hormone, and growth hormone (GH).

Testosterone has received great attention in recent years. From the pathophysiological viewpoint, in the presence of ventricular dysfunction, testosterone can modulate the cardiac remodeling process, strengthen skeletal musculature, enhance exercise capacity and reduce inflammatory activity.^5,6^ It is worth noting that a significant number of patients with heart failure have reduced testosterone levels. In addition, low testosterone levels in patients with heart failure are an independent risk factor for hospital readmission within 90 days and for mortality.^7^ Thus, testosterone supplementation seems an attractive strategy to manage heart failure.

In different clinical studies, testosterone supplementation improved the exercise capacity of patients with heart failure, regardless of their hormone levels. However, hormone treatment did not modify objective parameters of cardiac remodeling, such as left ventricular dimensions or ventricular function.^8^ Thus, the so far available evidence suggests that the beneficial effects of testosterone supplementation could derive from its preferential action on skeletal musculature rather than on cardiac remodeling.

Regarding thyroid hormone, different actions have been described, such as inotropic, chronotropic and lusitropic effects.^9,10^ In addition, remodeling-related actions, such as antiapoptotic, antiinflammatory and antifibrotic properties, angiogenesis promotion, cardiac regeneration and induction of beneficial micro RNA profiles, have been reported. Thus, changes in thyroid function affect cardiac morphology and function, in addition to being risk factors for the appearance of heart failure.^11,12^

Of the changes in thyroid function associated with cardiac remodeling, low T3 syndrome stands out, characterized mainly by an increase in the conversion of T4 to reverse T3, an inactive form of the thyroid hormone. That syndrome can be present in approximately 30% of patients with advanced heart failure, being an independent predictor of cardiovascular mortality. In addition, experimental studies have shown that low cardiac T3 levels can occur in the presence of normal serum levels of thyroid hormones. It is worth noting that cardiac aggressions, such as myocardial infarction, arterial hypertension and diabetes, can reduce the cardiac tissue levels of T3.^11-13^

That has supported the use of thyroid hormones to prevent or attenuate cardiac remodeling. In different experimental models of aggression, the administration of thyroid hormones was accompanied by an improvement in the cardiac cellular, morphological and functional variables. However, in humans, the scarce information available results from small studies assessing only inconsistent hemodynamic and functional effects.^11-14^

Although GH has gained special attention in the past years because of its claimed effect of delaying aging process and potential increase in physical performance, it is considered a modulator of cardiac morphology and function.^15^ Both excess and deficiency of GH and its mediator (insulin-like growth factor I - IGF-1) are associated with cardiovascular disease.

In different experimental models, GH administration was associated with a reduction in cardiac remodeling secondary to diverse stimuli.^16-18^ Approximately 30% of patients with heart failure have GH deficiency, and low IGF-1 levels were predictors of mortality.^8^ All those considerations support GH supplementation to patients with cardiac dysfunction. Although the first clinical studies have claimed beneficial effects of GH, two placebo-controlled studies have shown neutral results in patients with heart failure.^19,20^ Thus, the role played by GH in cardiac remodeling situations remains to be determined.

Despite the solid pathophysiological evidence and consistent experimental results, so far there is no evidence of the clinical benefit of the routine administration of testosterone, thyroid hormone and GH to patients with cardiac remodeling.
